# Interactive design modeling of 3D styling combining Bezier curve and Harris corner point detection algorithm

**DOI:** 10.1371/journal.pone.0319323

**Published:** 2025-04-16

**Authors:** Hua Song, Ziqiao Wen

**Affiliations:** 1 Cheung Kong School of Art and Design, Shantou University, Shantou, China; 2 School of Journalism Literature and Law, Wuchang Shouyi University, Wuhan, China; Wilfrid Laurier University, Canada

## Abstract

Enhancing the effectiveness and precision of 3D modeling design has become the main focus of current research due to the ongoing development of 3D modeling applications in numerous fields. In this study, an interactive 3D modeling design model based on Bezier curve and Harris corner detection algorithm is constructed. First, the output image is denoised, and then the improved Harris corner detection algorithm is used to obtain the feature points, which ensures the clarity of the image while extracting the key features. Second, a new sketch contour generation method is proposed by combining feature points with Bezier curves. Finally, a combination of Bezier surfaces and Delaunay triangulation is used for surface reconstruction and 3D modeling of the image is rendered. The results revealed that the improved Harris corner point detection algorithm could detect 120 3D image features of a kettle in 3.2s, and detect 50 3D image feature points of a vase in only 0.43s, which had very high detection speed and accuracy. The total time for surface reconstruction of a mechanical part model by combining Bezier surface and Delaunay triangulation was 0.524s, which met the basic user requirements. The teapot image modeling experiment proves the feasibility of the overall design method of the study. Moreover, through the 3D modeling interaction design model designed by the study, the designer can construct the accurate 3D modeling based on the real thing faster, which greatly saves time, improves efficiency, and pushes forward the development of 3D modeling design technology.

## 1. Introduction

Three-dimensional modeling (3DM) technology is becoming more and more common in a variety of disciplines due to the quick advancement of science and technology as well as the growing demands of society [1]. From mechanical modeling design to media animation production to intelligent manufacturing and other fields, 3DM plays an important role. In addition to offering a more natural and intuitive visual experience, it also facilitates deeper understanding and recognition of objects and entities [2]. Therefore, improving the efficiency and accuracy of 3DM design has become a focus of current research. At present, according to the actual situation of model design, 3DM design occupies a larger market. Many experts and scholars have also conducted relevant research. Nebot J et al. proposed a new product shape definition method for the limitations of traditional 3DM programs in terms of current manufacturing capabilities. This method was able to overcome the limitations of the design process and better exploit the enormous potential of modern manufacturing technologies, especially additive manufacturing. However, the proposed approach remained purely speculative [3]. Hu L et al. mainly focused on the problems in the design of engineering drawings of 3D solid models, and proposed the method of using 3D CAD software to create an “orthographic projection digital model of the object”. Through a comprehensive analysis and comparison of various plastic part feature classification schemes, a 3D feature library of injection molded products was established using 3D feature modeling technology and parametric technology. The results showed that the method could well demonstrate the object 3D physical model [4]. Jing Y et al. summarized the effective method of shaping 3D animation character model construction through the author’s own creative practice for small-scale 3D animation companies in the case of relatively weak technology [5]. A paradigm was put out by Ban S. et al. to solve the difficulties associated with the exploration process during the early stages of 3D model design. The framework interpolated input designs and sketches from a database to explore unexplored design combinations. It then developed an interactive 3D model reconstruction technique that enabled shape alterations of design elements. The results indicated that more and higher quality 3D model designs could be generated faster using the proposed framework [6]. Cheng X et al. artificially realized the transformation of 2D films into programmable 3D curves through mechanically guided assembly. The heterogeneous 2D microstructural patterns required to realize the target 3D curves were identified through analytical modeling and machine learning based computational methods. The results of the study presented about 30 different 3D curve morphologies [7]. Liu Y et al. proposed an industrial interactive design system based on 3D technology to address issues related to industrial robot design. This system combined the functions and schematic design of industrial robot assembly and adjustment, and used 3DM tools (such as SolidWorks and 3DsMAX), VR development engine (Unity 3D), and VR device (HTC VIVE) for implementation. The results showed that this 3D interactive design technology could provide a more intuitive and interactive learning experience in the field of education[8]. Adisusilo A et al. aimed to provide an immersive and interactive way of 3DM design. A 3D interactive modeling design was constructed using Unity 3D platform, C # programming algorithm, finite state machine (FSM) to design a player control system, 3D Blender to create 3D objects, and other technologies. The results showed that the 3D interactive modeling design of the campus had a high degree of immersion and interactivity [9].

In the existing 3DM design process, model sketching is an essential part. Nevertheless, the conventional hand-drawing approach has the drawback of blurring drawings as a result of repeated erasures, while commercial sketching software frequently contains difficult-to-use commands. Furthermore, an inefficient design is produced when the accuracy of fitting the 3DM design grows along with an increase in image processing. In this research, Hayat et al. developed a novel framework based on deep convolutional neural network (DCNN) in response to the rising popularity of hand-drawn sketch detection and retrieval. Three popular pre-trained DCNN architectures using the global average pooling (GAP) technique were used by the framework in the transfer learning setting. It was discovered that the suggested framework significantly improved the classification and retrieval of image sketches [10]. Manda B et al. proposed a deep learning (DL) approach to improve sketching for the problem of model sketching in engineering. First, various types of sketches were analyzed for defects to create cleaned or enhanced query sketch datasets. Subsequently, end-to-end deep neural network training was performed to establish mapping relationships between defective sketches and cleaned/enhanced sketches. It was found that the methodology used in the study significantly improved the quality of the sketches [11]. In response to the issue that previous sketch-based 3D model retrieval efforts have overlooked the dynamic features of the data, Bai J et al. suggested an end-to-end 3D model sketch retrieval approach based on joint embedding of spatio-temporal information. The method characterized sketches as dynamic drawing sequences while 3D models as multi-view sequences. The results revealed that the methodology used in the study has high accuracy [12]. Zhu Z et al. proposed a fast 3D geometry simplification method called Alpha-SIM to address the time-consuming and error-prone problem of modeling electromechanical and pneumatic wiring interconnect systems. The method generated 2D profiles of point projections by controlling the density and Alpha value of 3D points to achieve different levels of geometric approximation. The method’s efficiency and effectiveness in simplifying geometric sketches for automated EWIS routing were demonstrated by experimental findings [13].

Nowadays, the application of 3DM interaction design (3D-MID) modeling is getting wider and wider, covering many fields. Arias-Rosales A et al. proposed a generative tool called “Albatros Create” for 3DM of wind turbine blade shapes. With the use of infographics, centralized parameterization, and interactive visualization, the tool was created to assist in the definition of aerodynamic curve geometries for horizontal axis turbines. Experimental results indicated that Albatros Create was able to generate smooth, fully editable 3D model curves compared to other software [14]. Guima K E et al. proposed a complete series of solutions from modeling to post-processing for 3D printing using fused deposition modeling (FDM). The results of the study showed that this method was effective in producing the desired 3D printed parts [15]. Okura F et al. 3DM and tree reconstruction using computer vision and graphics. To achieve 3DM construction of plants and trees, each plant modeling and reconstruction approach was based on shape/structure representations, which were first summarized [16].

After a summary of the studies conducted by the aforementioned researchers, it can be said that 3D-MID is significant in today’s social life. However, existing 3D design models generally suffer from high computational complexity, insufficient accuracy, and poor visualization. To address these challenges, 3D-MID is developed as an innovative method. The method cleverly combines Bezier curves and Harris corner detection algorithm (HCDA), aiming to achieve fast and accurate design of 3D image modeling. The introduction of this novel methodology is anticipated to facilitate a substantial advancement in the domain of 3D design, enhance the design experience for users, and facilitate the advancement and application of related technologies.

The innovation of the research lies in the construction of an interactive 3D modeling design model (3D-MID) based on Bezier curves and Harris corner detection algorithm. This model quickly and accurately identifies image feature points by improving the Harris corner detection algorithm, generates smooth sketch contours using Bezier curves, and combines Bezier surfaces and Delaunay triangulation for surface reconstruction, achieving efficient and accurate 3D modeling. Compared to traditional methods, this model not only improves modeling accuracy and stability, but also significantly reduces computational complexity and enhances design efficiency. In addition, the model has high interactivity, allowing designers to modify and adjust based on identified feature points, making the design process more flexible and better meeting the diverse needs of designers and clients. The results of this research have shown great potential in high-precision, high-efficiency, and highly interactive 3D modeling fields such as product design, virtual reality, and medical image reconstruction, providing important impetus for promoting technological innovation and industrial upgrading in related fields.

There are five primary sections of the study. The first section leads into the research design technique by primarily discussing the state of 3DM technology and model development. The unique building process of the 3D-MID model is mostly introduced in the second section. The third part mainly designs the experimental environment and verifies the effectiveness of the algorithms designed in the 3D-MID model building and the design effect of the model. The fourth part mainly discusses the main results of the study. The fifth part is to analyze the experimental results and elaborate the shortcomings of the research method.

## 2. Methods and materials

This section outlines the 3D-MID model building method. The image denoising method and feature point (FP) extraction method when constructing the 3DM, the drawing method of contour curves, the reconstruction method of model curves and the rendering method are elaborated.

### 2.1. 3DM interaction design model building

**Fig 1:**
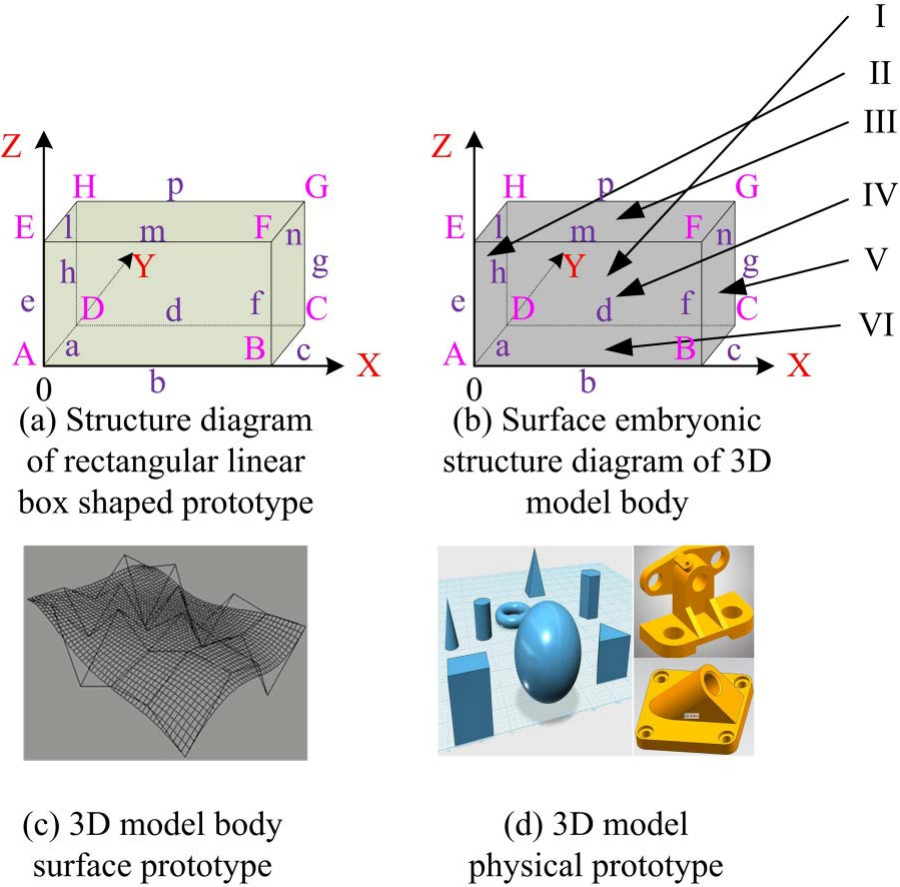
Schematic of the prototype structure of the base model.

To build a 3D-MID model, it is necessary to first build a prototype of the basic model, including a linear box shaped prototype, a 3D model body surface prototype, a 3D model curved surface prototype, a 3D model physical prototype, and a 3D model features prototype [17]. The representation methods for each prototype are shown in Fig 1.

In Fig 1, when building the 3D-MID model, the linear box shaped prototype mainly realizes the preservation of the entire wireframe prototype by recording the positions of the vertices and edges of the cube. The 3D model body surface prototype is mainly realized by recording the relative positions of the faces and tables of the entire wireframe on the basis of the previously saved wireframe prototype. The 3D model curved surface prototype is to construct the curved piece first, and then construct the curve prototype by stitching and assembling the curved piece and save it. The construction of 3D model physical prototype mainly adopts scanning method and voxel method, in which the scanning method is to construct the physical prototype in three-dimensional space by rotating and flattening the closed curved contour on the plane. The voxel method is to use the basic voxel information that comes with the computer 3DM software to construct the physical prototype. 3D model features prototype is to add the characteristic features of the constructed model on the basis of the physical prototype to make the model more specific [18].

3D-MID modeling also requires sketching, and the most important part of the sketching process is the production of “splines”. The most important part of the sketching process is the production of “splines”. The curve production in the “spline” production process adopts the interpolation method, interpolation is a mathematical method that constructs a curve through a set of known data points using a certain algorithm or formula. This curve is called an interpolation curve. Setting $P_{i}\left(i=0,1,2...,0\right)$ as a set of data points, and the curve constructed through this set of data points is set as the interpolation curve. Its function expression is Equation (1) [19].


$$f\left(x\right)=y_{j-1}+\frac{\left(x-y_{j-1}\right)\left(y_{i}-y_{j-1}\right)}{x_{j}-x_{j-1}}$$ (1)

Where, *x* is the interpolation in $\left[x_{j-1},x_{j}\right]$. $\left(x_{i},y_{j}\right)$ is the curve coordinates. The value range of *u*is$x_{j-1}\leq u\leq x_{j}$. Setting the numerous function values of the function $f\left(x\right)$as$\left\{f_{1}\left(x\right),f_{2}\left(x\right),...,f_{n}\left(x\right)\right\}$, the study uses the method of adjusting the coefficients to be determined to reduce the gap between the known points in the sketch and the curve function, and then uses the method of segmentation to process the data points step by step and obtain the desired data curve. The study takes the construction of B spline curve (SC), which is commonly used in sketching, as an example for illustration. In sketching, the desired control point (CP) positions of the whole sketch are set and then the B-spline basis function (B-SBF) linear combination is selected. The B-SBF is shown in Equation (2) [20].


$$\{\begin{array}{c} N_{u,0} \{\begin{array}{c} h_{u}\leq h\leq h_{u+1}\\ 0 \end{array}\\ N_{u,v}\left(h\right)=\frac{h-h_{u}}{h_{u+v}-h_{u}}N_{u,v-1}\left(h\right)+\frac{h_{u+v+1}-h}{h_{u+v+1}-h_{u+1}}N_{u+1,v-1}\left(h\right) \end{array}$$ (2)

Where, *h*is a single parameter and$h_{u}$,$h_{u+1}$,$h_{u+v}$,$h_{u+v+l}$, etc. are the nodes of B-SBF. *u*in $N_{u,v}\left(h\right)$is the ordinal number of the function and *v*is the times of the function. $\left[h_{u},h_{u+v+l}\right]$is the construction range of$N_{u,v}\left(h\right)$, within which the modification of the curve is realized by moving the spline base fall. Connecting the set CPs with the desired curve is SC, assuming that the serial number of a certain set of CPs is$\left\{h_{u}\right\}$, and then define the vertex $\left\{p_{u}\right\}$to form the polygon of B-SBF$N_{u,v}\left(h\right)$. By combining $N_{u,v}\left(h\right)$and$\left\{p_{u}\right\}$, the SC equation of *v*th v+1order can be derived as shown in Equation (3).


$$\{\begin{array}{c} r\left(h\right)=\sum_{u=0}^{n}N_{u,v}\left(h\right)p_{u}\\ m\leq h\leq n \end{array}$$ (3)

Where, $r\left(h\right)$is the *v*subsegment polynomial of*h*. Also define another set of data points $\{\begin{array}{c} X=\left\{X_{j}\right\}\\ j=1,2,3,4,...,n \end{array}$that coincide with the set B SC, which satisfies Equation (4).


$$X_{j}\left(h_{j}\right)=\sum_{j=0}^{n}N_{j,v}\left(h\right)P_{j}\begin{array}{cc} & j=1,2,... \end{array},n$$ (4)

**Fig 2:**
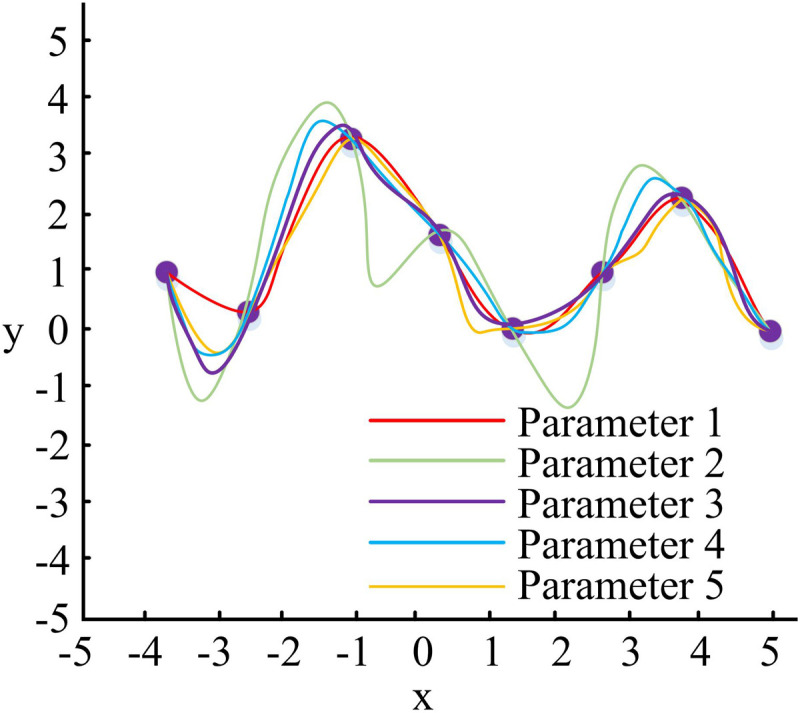
Schematic diagram of spline curve adjustment.

Equation (4) is converted to the X=NPform to represent it. Then parameterize *X*and set the specific position of the CP. The control node vertices and matrix *N*of the B-SBF can be solved. The SC adjustment is shown in Fig 2.

The horizontal axis *x* and vertical axis *y* in Fig 2 represent the distance in the horizontal and vertical directions of the Cartesian coordinate system, respectively. Fig 2 lists the adjustment scheme of five parameters. To achieve accurate control over the model contour, the SC drawing in the sketch progressively modifies the curve’s shape based on the identification of the CPs.

### 2.2. Construction of image contours based on Bezier curves with HCDA 3DM

The previous section has described the method of sketching model contours using SC, while before model contour generation, it is necessary to first construct the Bezier basis function and then input the relevant image information. Let the multinomial CPs of the Bezier basis function be$B_{0,3}\left(t\right)$,$B_{1,3}\left(t\right)$, $B_{2,3}\left(t\right)$and$B_{3,3}\left(t\right)$. The expressions of the four multinomial CPs are shown in Equation (5).


$$\{\begin{array}{c} B_{3.3}\left(t\right)=3t^{3}\\ B_{2.3}\left(t\right)=3t^{3}\left(1-t\right)\\ B_{1.3}\left(t\right)=3t{\left(1-t\right)}^{2}\\ B_{0.3}\left(t\right)={\left(1-t\right)}^{3} \end{array}$$ (5)

Where, *t*is the horizontal coordinate of the multinomial CP. Let$P_{0}$,$P_{1}$,$P_{2}$, $P_{3}$be the monomial CPs of the Bezier basis function. Then the Bezier curve expression $Q\left(t\right)$can be obtained, as illustrated in Equation (6).


$$Q\left(t\right)=\sum_{i}^{3}P_{i}B_{i,3}\left(t\right)$$ (6)

Bezier curves define the shape of the curve by controlling the position and weight of CPs, focusing more on generating contours that meet design requirements directly through mathematical formulas and calculations. The traditional curve fitting technique is based on a series of known data points and constructs a curve through these data points using a specific model. As a result, Bezier curves are more appropriate for design areas that require precise control of contour shape and detail. Traditional curve fitting techniques are more appropriate for fields that require extracting useful trends and information from large amounts of data [21].

The study uses Bezier curves to create the contours of the model, which are adjusted and modified to draw 3DM images (3DMI)) according to the target requirements of 3DM design. The 3D model contours created through Bezier curves often have complex shapes and details. During the image input process, these details may become blurred due to noise interference. To ensure the overall accuracy and clarity of the image input, an adaptive median filtering algorithm is investigated to denoise the output image [22]. Equation (7) illustrates the first layer of the two-part AMF algorithm.


$$\{\begin{array}{c} Z_{A1}=Z_{med}-Z_{\min}\\ Z_{A2}=Z_{\max}-Z_{med} \end{array}$$ (7)

Where, if $\{\begin{array}{c} Z_{A1}>0\\ Z_{A2}>0 \end{array}$, then go directly to layer 1. If$\{\begin{array}{c} Z_{A1}<0\\ Z_{A2}<0 \end{array}$, the image window is increased. In addition, if window size$\leq Z_{\text{max}}$, go to layer 1 and repeat the operation. If window size$\geq Z_{\text{max}}$, output pixel value$Z_{med}$. The 1st layer is shown in Equation (8).


$$\{\begin{array}{c} Z_{B1}=Z_{\text{xy}}-Z_{\min}\\ Z_{B2}=Z_{\max}-Z_{\text{xy}} \end{array}$$ (8)

**Fig 3:**
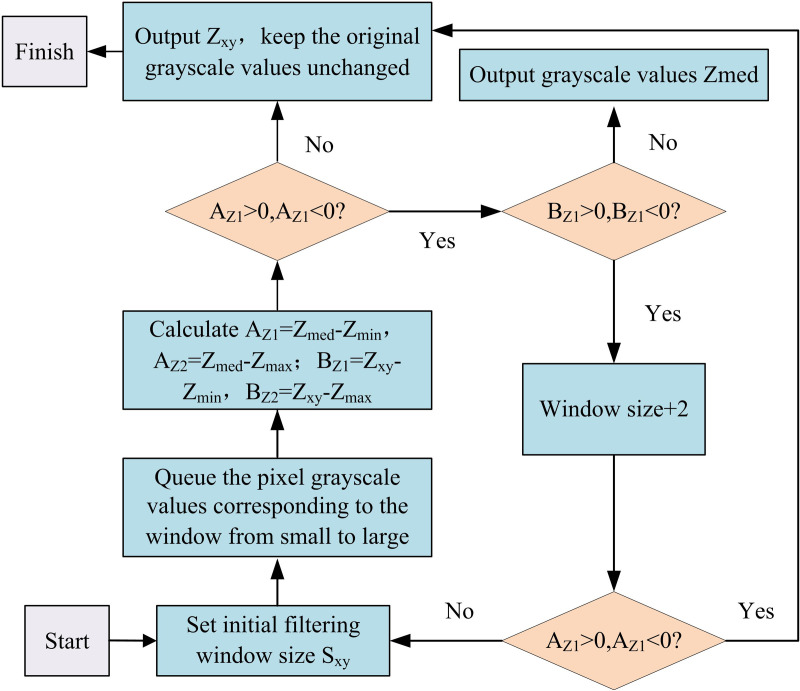
Adaptive media filtering algorithm flowchart.

Where, if$\{\begin{array}{c} Z_{B1}>0\\ Z_{B2}>0 \end{array}$, the pixel value $Z_{xy}$is output. If$\{\begin{array}{c} Z_{B1}<0\\ Z_{B2}<0 \end{array}$, the pixel value $Z_{med}$is output. In the variables of the above two equations, $S_{\min}$and $S_{\text{max}}$are the maximum and minimum values of the image window size, respectively.$Z_{\min}$, $Z_{\text{max}}$and $Z_{med}$are the minimum, maximum and median values of the gray level of the pixel in the considered window, respectively. $Z_{xy}$is the gray value at the image pixel point$\left(x,y\right)$. In summary, the AMF algorithm denoises the drawn 3DMI by adaptively adjusting the window size to get the optimal size of the consideration window. The whole denoising process is shown in Fig 3.

Nevertheless, the study additionally presents the discrete wavelet transform to denoise the picture operation in order to further enhance the clarity and accuracy of the produced 3DMI. Using the algorithm in Fig 3 alone is by no means sufficient to achieve this goal. The specific program is to carry out three-layer discrete wavelet transform operation on the image denoised by the AMF algorithm to obtain the high and low-frequency decomposition coefficients (DCS) of the 3DMI. For the denoising of high frequency DCSs image, the study uses wavelet soft threshold denoising function model for denoising, as shown in Equation (9).


$$\{\begin{array}{c} \hat{\omega }= \{\begin{array}{c} \begin{array}{cc} \mathrm{sgn}\left(\omega \right)\left(\left|\omega \right|-2^{2-l}•t\right) & \left|\omega \right|\geq t \end{array}\\ \begin{array}{cc} 0 & \left|\omega \right|<t \end{array} \end{array}\\ t=\frac{median\left\{\left|\omega \right|\right\}\sqrt{2\ln\left(X\times Y\right)}}{0.6745} \end{array}$$ (9)

Where, *ω*is the wavelet coefficients and $\hat{\omega }$is the original wavelet coefficients. *t*is the wavelet threshold and *l* is the number of wavelet decomposition layers. medianis the median calculation method, *X*and *Y*are the image sizes waiting for denoising. Finally, the original low-frequency DCSs and the denoised high-frequency DCSs are reconstructed, and the image with higher clarity and accuracy can be obtained. While the integration of an adaptive median filtering algorithm with a discrete wavelet transform can markedly enhance the clarity and precision of 3DM images, it will concomitantly elevate the complexity of the system. Therefore, by reducing the number of wavelet decomposition layers to sacrifice some image quality, the complexity can be reduced. The computational efficiency is inversely proportional to the image quality. The higher the image quality and the more complex the model, the lower the computational efficiency. The 3D modeling interactive design model constructed by the research prioritizes image quality, with a set image quality resolution of 2560 × 1440. The complexity of the model is adjusted accordingly based on the image quality.

After denoising the 3DMI again, the study then uses the improved HCDA to extract the FPs of the image, which improves the efficiency of the 3DMI sketch outline [23]. The traditional Harris corner detection algorithm is based on the gradient of pixel gray value changes in the image, and its key process is divided into 5 steps: 1. Convert the original image into a grayscale image. 2. Use differential operators to calculate the gradients of the image in both horizontal and vertical directions. 3. Apply Gaussian smoothing to gradient images to remove noise and unnecessary detail. 4. Use the Corner Response function to calculate the corner response value of each pixel and find the local maximum. 5. Confirm corner points: Set a threshold and identify points with response values greater than the threshold and local maxima as vertices. The specific method is as followed: It is assumed that the output 3DMI*f*, $\left(x,y\right)$is the window center coordinates, $\left(m,n\right)$is the coordinates of the window center image, and $V_{m,n}$is the Gaussian function. The *f*is first grayed out and then translated and the autocorrelation function $Q\left(x,y\right)$is obtained as shown in Equation (10).


$$Q\left(x,y\right)=\sum_{m,n}V_{m,n}{\left|f\left(x+m\right),f\left(y+n\right)-f\left(x,y\right)\right|}^{2}$$ (10)

The eigenvalues of the quadratic function are used to transform Equation (11) into a matrix expression, as shown in Equation (11).


$$Q\left(x,\;y\right)=\sum_{m,n}\left[\left[x,\;y\right]\begin{array}{cc} f_{m}^{2} & f_{m}f_{n}\\ f_{m}f_{n} & f_{n}^{2} \end{array}{\left[x,y\right]}^{T}\right]$$ (11)

Both $f_{m}$and $f_{n}$in Equation (12) are partial derivatives of$f\left(x,y\right)$. HCDA mainly uses the corner point (Cor-P) response value function to judge the Cor-Ps in the image, the Cor-P response value function*R*, as shown in Equation (12).


$$R=\det\left(Q\left(x,y\right)\right)-k•trace^{2}\left(Q\left(x,y\right)\right)$$ (12)

**Fig 4:**
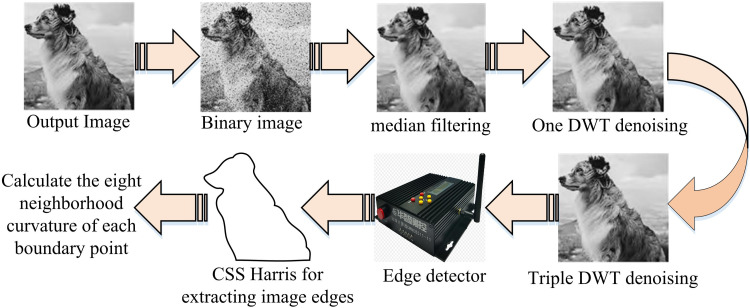
Flowchart of CSS-Harris detection algorithm to extract image feature points.

Where, $\det\left(Q\left(x,y\right)\right)$is the determinant of the matrix, *k*is the coefficients and $trace\left(Q\left(x,y\right)\right)$is the trace of$Q\left(x,y\right)$. By introducing CSS (Curvature Scale Space Corner Detector) technology, the CSS Harris detection algorithm can more accurately extract FPs in images, reducing false positives and false negatives [24]. Fig 4 depicts the stages of the CSS-Harris detection algorithm used to extract the image FPs.

The study specifies 0 and 255 as the intensity values of the non-edge pixels and the edge pixels of the detected picture, respectively, in Fig 4, when the image is detected using the edge detector. In the final calculation of the curvature of the boundary points of the image, the CSS detection technique is used to obtain the image I. The result is then applied to the Harris algorithm to obtain the image II, and I and II are combined to obtain the corner candidate set III, which is then based on the corner candidate set to determine the FPs of the image. The investigation of image FP tracking aims to prevent the issue of omission in image FP detection. The starting point of tracking, designated as*M*, is determined by taking the detected image’s maximum gray value. Taking *M*as the starting point, another point with the highest gray value is searched for as the subsequent FP in the eight-neighborhood of the point, defined as*N*. The above operation is repeated with *N*as the starting point until the conditions are finally met. In this manner, the image’s border points can be located, and tracking of the identified FPs can be accomplished. In summary, the combination of Bezier curve Harris corner detection algorithm is to first use Bezier curve to create a preliminary contour of the model according to the requirements, and adjust the position and weight of control points to finely control the shape and details of the contour. Then, the Harris corner detection algorithm is used to extract feature points from the image. Apply the extracted feature points to the control point adjustment of Bezier curves to further optimize the shape and details of the contour.

### 2.3. 3DM image curve construction based on contour curves

Bezier curves are further constructed on the basis of sketched contour Bezier curves to complete the 3DM design of the image. The Bezier curve is composed of a number of Bezier curve slices, and the study sets a Bezier curve slice is composed of 5 ×  5 CPs. The Bezier curve slice function is shown in Equation (13).


$$\{\begin{array}{c} Q\left(u,v\right)=\sum_{j=0}^{3}\sum_{i=0}^{3}P_{i,j}\left(u\right)B_{i,3}\left(u\right)B_{j,3}\left(v\right)\\ 0\leq u\leq 1\\ 0\leq v\leq 1 \end{array}$$ (13)

**Fig 5:**
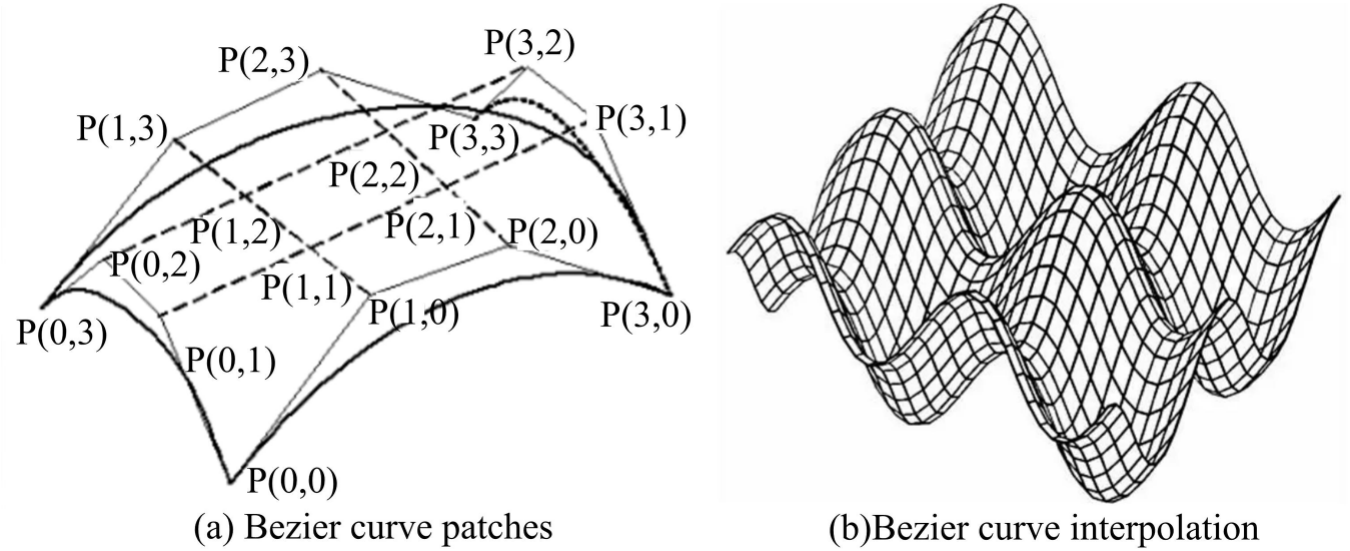
Bezier curve patches with BCI.

Where, *v* and *u* are variables. $P_{i,j}\left(u\right)$ is the basic shape outline of the Bezier curve patches. $B_{i,3}$ and $B_{j,3}$ are CPs. The CPs are interpolated together using interpolation to obtain the interpolated curve, and the contour line of the interpolated curve is the Bezier curve [25]. The process of transforming from a Bezier curve patches to an interpolated curve is shown in Fig 5.

**Fig 6:**
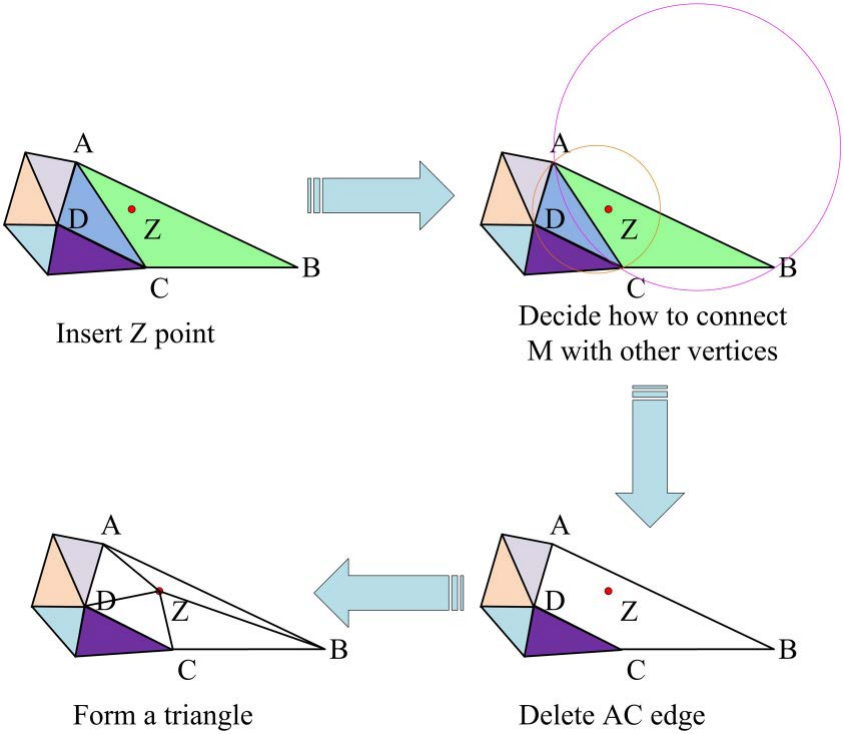
Delaunay triangulation process.

As shown in Fig 5, a number of Bezier curve patches can be combined to form different shapes of Bezier curve interpolation (BCI), and then the BCI is subjected to Delaunay triangulation, and the specific flow is shown in Fig 6.

As shown in Fig 6, first define a CP *Z* in the BCI; then select a larger triangle containing CP *Z*, and place it in the triangle chain table for saving; then find the outer circle of the triangle containing CP *Z* in the triangle chain table; after that, delete the common edges of the outer circle, and connect the CP *Z* with other vertices, to complete the Delaunay triangulation. In summary, the curve reconstruction process is achieved by constructing Bezier surface patches defined by 5 ×  5 control points based on existing sketch outline Bezier curves. These control points are used to interpolate and generate continuous interpolated surfaces, where the shape of each Bezier surface patch is determined by the corresponding control point mesh. By combining multiple such Bezier surface patches, complex and varied Bezier interpolation surfaces can be formed, thereby completing the 3D modeling design of images. Subsequently, the constructed Bezier interpolation surface is subjected to Delaunay triangulation. By defining control points, selecting larger triangles containing control points, finding circumcircles, and reconnecting vertices, the surface is finely segmented and optimized, providing a foundation for subsequent 3D modeling and rendering.

The curve is then rendered by setting the type-valued point of the curve to be $P_{i}\left(i=0,1,2...n\right)$and the tangent vector of the type-valued point of each curve to be$P_{�}^{i}\left(i=0,1,2...n\right)$. The expression for the slice of the curve is given in Equation (14) [26].


$$\{\begin{array}{c} R_{i}\left(u\right)=P_{i-1}F_{0}\left(u\right)+P_{i}F_{l}\left(u\right)+h_{i}\left[P_{i-1}G_{0}\left(u\right)+P_{�}^{i}F_{l}\left(u\right)\right]\\ 0\leq u\leq l\\ l\leq i\leq n \end{array}$$ (14)

Where, *F*and *G*are mixing functions and $P_{i-1}^{,}$and $P_{i}^{,}$are denoted as tangent vectors. According to the mechanical theory, the bending performance index of the curve is shown in Equation (15).


$$U=\frac{EJ}{2}\int_{0}^{l}k^{2}\left(s\right)ds$$ (15)

Where, *l* is the spline length of the curve, *s*is the arc length. $k\left(s\right)$is the curvature where the arc length position is located, and EJrefers to the bending strength. The curvature at the position of the arc length is shown in Equation (16).


$$k\left(s\right)=\frac{l}{h_{i}^{2}}R_{i}^{,,}\left(u\right)$$ (16)

Where, *i* is the number of slices of the curve. The bending elastic energy index of the *i* th slice is $U_{i}$, as shown in Equation (17).


$$U_{i}=\frac{EJ}{2h_{i}^{3}}\int_{i}^{,,2}\left(u\right)du$$ (17)

The total elasticity index for the entire curve slice of the SC is shown in Equation (18).


$$U=U_{1}+U_{2}+U_{3}+U_{4}...+U_{n}$$ (18)

From the above equations, it can be concluded that the smooth rendering of 3DMI curve is related to the piecewise type value points and tangent vectors. Therefore, the rendering of 3DMI curve is realized by calculating the piecewise type value points and tangent vectors to satisfy the customer’s demand. When dealing with more complex geometric shapes or noisy environments, current rendering techniques may encounter some potential limitations. This study uses the Catmull-Rom subdivision method in subdivision surface technology to produce smoother and more detailed surfaces. New vertices are generated on the original mesh by interpolation or approximation, thereby refining the mesh and improving the continuity of the surface. When dealing with noisy data, Gaussian filtering is used to reduce the impact of noise on rendering results. The Gaussian function is shown in Equation (19).


$${\text{Pixel\_f(x,y) = (1/(2πσ}}^{\text{2}}{\text{))}}^{\text{0.5}}{\text{* exp(-(x}}^{\text{2}}{\text{+y}}^{\text{2}}{\text{)/(2σ}}^{\text{2}}\text{))}$$ (19)

Where, $\left(x,y\right)$is the coordinate of the pixel point. *δ*is the standard deviation of the Gaussian function, which determines the smoothness of the filtering. The Gaussian function is first discretized to obtain an N × N filtering kernel matrix. Then, the filtering kernel is convolved with the 3DM image, i.e., for each pixel point, the gray value of the surrounding pixels is multiplied by the weight of the corresponding position in the filtering kernel and summed to obtain the filtered gray value of the pixel point. Finally, the filtering strength is adjusted based on the local characteristics of the 3DM image to remove noise while preserving detail.

In summary, the 3D-MID method constructed by the research is specifically as follows: the image to be modeled is input, and then the image is denoised using median filtering and DWT denoising algorithms. Then, the Bezier basis function is utilized to generate the cubic Bezier curve, and the outline of the closed sketch is outlined by means of FP interpolation. The sketch design is then sent to the necessary staff member or designer for assessment to make sure the design satisfies the specifications. Finally, the curve reconstruction is completed with the help of triangulation technique based on Bezier curve, and the curve of the model is rendered by using the curve rendering method based on slicing, so as to satisfy the customer’s aesthetic needs and visual requirements.

## 3. Results

In this section, the experimental environment is designed to evaluate the effectiveness of the denoising algorithm, feature extraction algorithm, and curve reconstruction algorithm used in constructing the 3D-MID model, and the effect of practical application of the 3D-MID model is evaluated.

### 3.1. 3DM interaction design modeling algorithm performance evaluation

#### 3.1.1. Performance evaluation of denoising algorithms.

The experiment’s environment is initially constructed by the research and is displayed in Table 1 in order to verify the efficacy of the algorithms employed for 3D-MID model development.

**Table 1:** Algorithm testing environment settings.

Project	Parameter
Central processing unit	Cortex-M
Data storage	MySQL data bank
The overall implementation platform of the system	Simulink
System PC side memory	32G
CPU dominant frequency	2.60GHz Core(i5)
GPU	GTX 1660 Ti
operating system	Windows7
Operating environment	MATLAB

**Fig 7:**
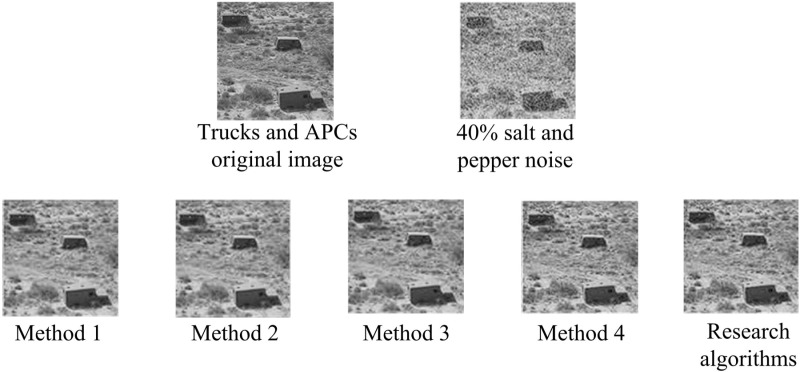
Results of different denoising algorithms for processing trucks and APCs model plots.

Computing resources are the hardware capabilities required to support the execution of complex algorithms and the efficient processing of large datasets, such as processing power (CPU) and memory. The Harris corner detection algorithm in this method requires gradient calculation, response value calculation, and corner extraction for each pixel in the image, all of which involve a large number of matrix operations and eigenvalue analysis, and thus have high computational complexity. Bezier curve drawing requires complex mathematical calculations. Especially in 3D space, curve generation and rendering require a significant amount of computational resources. The adaptive median filtering, third-order discrete wavelet transform denoising, and CSS enhanced Harris feature detection algorithms interspersed throughout the process increase the overall computational load. Therefore, in the hardware approach, a 2.60GHz Core (i5) processor is selected as the basis for executing complex algorithms. The 32GB of memory is chosen to ensure stable system operation when processing high-resolution images and large amounts of data. Firstly, the efficacy of the denoising techniques employed in the study is evaluated. The model map of trucks and APCs is selected as the experimental subject, with 40% of the image corrupted by a predefined noise. The model map is then denoised using the AMF algorithm (Method 1), an image denoising algorithm with a median filter, and the results are compared. Additionally, the fractional-order filter (Method 2), full-frequency image denoising algorithm with multiple-head attentional mechanism (Method 3), image denoising algorithm with improved three-dimensional block-matched filtering (Method 4), as well as research algorithms, are evaluated. The specific results are presented in Fig 7.

**Fig 8:**
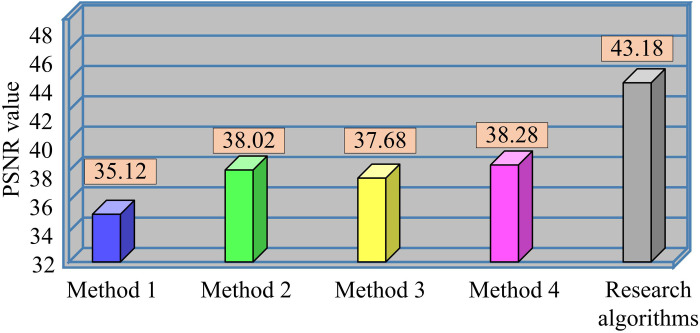
PSNR values for different denoising algorithms.

With the method under study, the noise in Fig 7 can be fully removed, and the image’s borders can be greatly improved, contributing to an even better visual effect. In contrast, after processing with median filtering, although it is able to reduce the noise, it still leaves some residual noise, which seriously affects the overall perception of the image. In addition, for the other algorithms, their enhancement effect is not obvious when dealing with the texture details of the image, and they cannot effectively highlight the features of the image. These observations indicate that the proposed algorithm of the study has higher processing efficiency and enhancement effect compared to other algorithms. It can better improve the image quality and make the image clearer and more visually appealing. To quantify the effectiveness of various image denoising algorithms, the study introduces the PSNR value to evaluate the denoising effect of various algorithms. One of the most used picture objective evaluation measures for comparing the denoised image’s difference from the original image is the PSNR value. The image quality improves with a higher PSNR value and a smaller disparity between the two. Fig 8 presents the test findings.

**Fig 9:**
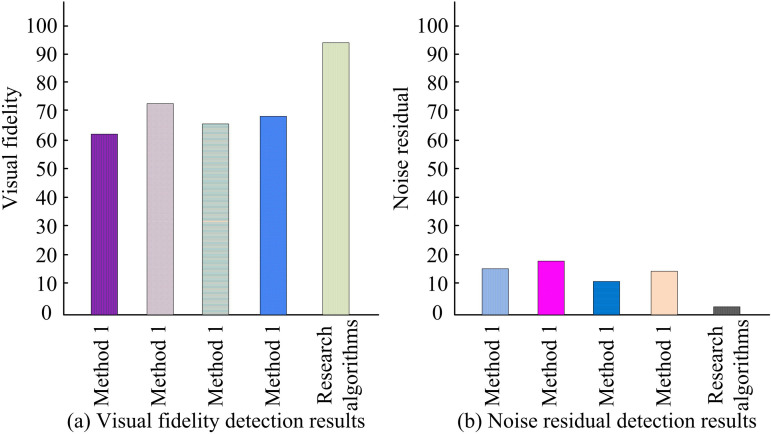
Visual fidelity and noise residual detection results of different denoising algorithms.

In Fig 8, the PSNR values of Method 1, Method 2, Method 3, Method 4 and the research method are 35.12, 38.02, 37.68, 38.28 and 43.18, respectively. Method 1 is able to dynamically adjust the size of the filter according to the local features of the image, so as to remove the noise efficiently, but the denoising accuracy is not very high when dealing with complex textures and high-noise images. Method 2 combines the advantages of median filtering and fractional-order filtering, which can better preserve image details and reduce the influence of noise, and thus obtains a relatively high PSNR value. Method 3 utilizes a multi-head attention mechanism to adaptively capture important features in the image, but the limitations of the attention mechanism result in slightly lower PSNR values than the other algorithms. Method 4 introduces a more effective 3D block matching strategy, which is able to recognize relevant information in the image more accurately, resulting in higher PSNR values. The denoising method used in the study, on the basis of AMF, is followed by three times wavelet denoising, so it possesses the highest PSNR value among similar denoising algorithms. Comparing the visual fidelity and noise residue of each model, the results are shown in Fig 9.

In Fig 9(a), the visual fidelity of Method 1 is 61%, indicating that approximately 61% of the original visual information is preserved in the processed image. The visual fidelity of Method 2 is improved to 72%, demonstrating its advantage in preserving image details. The visual fidelity of Method 3 is 64%, which is slightly higher than Method 1 but still lower than Method 2. The visual fidelity of Method 4 is 66%, which is slightly higher than Method 3 but does not reach the level of Method 2. The visual fidelity of the research method is as high as 93%, far surpassing all other methods, proving the excellent performance of the research method in preserving the visual information of images. According to Fig 9(b), the residual noise of Method 1 is 15%, indicating that a considerable amount of noise has not been effectively removed during the denoising process. The residual noise level of Method 2 is 18%, which is slightly higher than Method 1. Nevertheless, in light of the enhanced visual fidelity, this augmentation is within an acceptable range. Method 3 has a low residual noise level of 10%, indicating its strong ability in denoising. The residual noise level of Method 4 is similar to that of Method 1, at 15%. The residual noise of the research method is only 3%, which is the lowest among all methods, further proving its excellent denoising effect.

#### 3.1.2 Performance evaluation of feature extraction algorithms.

The experimental setting for testing is the same as in the previous section, which first verifies the effectiveness of the CSS techniques cited in the study for the improvement of the Harris feature extraction algorithm. The maximum and minimum pixel grayscale values for each algorithm’s image window is set to 800 × 800 and 600 × 00, respectively.

**Fig 10:**
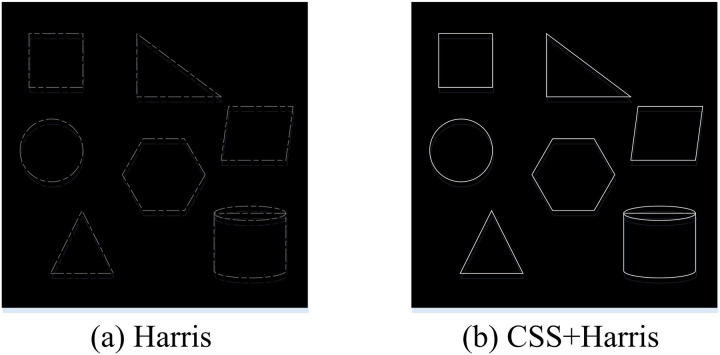
Feature point extraction effect of Harris feature extraction algorithm and its improved algorithm.

**Fig 11:**
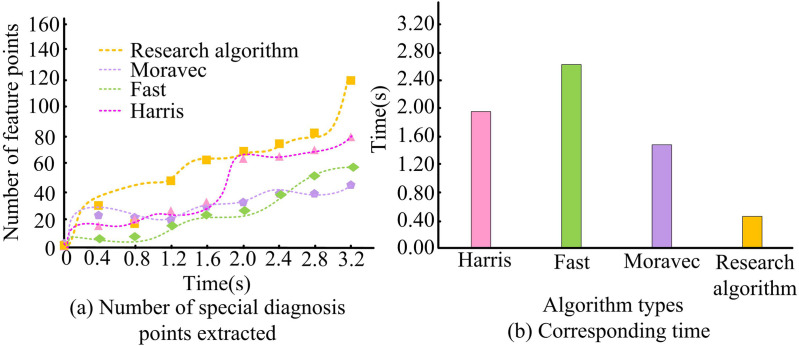
Comparison of feature detection speeds of different algorithms.

Fig 10(a) shows the processing results of Harris algorithm after extracting the FPs of some 3DM base image contour curves. The image contour FP extraction with only Harris algorithm is poor, the contour curve is not clear and the FPs are missing in many locations. Conversely, in Fig 10(b), the points extracted by CSS+Harris algorithm are more able to describe the information of the image more clearly, and the contour curve of the image is very clear and the FPs are prominent. The effectiveness of CSS technique to improve Harris algorithm is verified. To facilitate a meaningful comparison, the results of similar feature extraction algorithms and research algorithms are selected for comparison. The algorithms selected for comparison are the Harris, Moravec, and Fast algorithms. The key parameters involved in Harris algorithm are threshold and window size. The threshold is used to determine which corners are considered valid, while the window size affects the locality of corner detection. The Moravec algorithm also depends on parameters such as window size and threshold. The window size determines the considered neighborhood range, while the threshold is used to filter corners. The window size of these two algorithms is consistent with the algorithm constructed by the research institute, set to 7x7, while the threshold size is set to 0.04 for both algorithms. The Fast algorithm mainly involves two parameters: the threshold for non maximum suppression and the number of consecutive pixels that need to be detected. Based on experience, the threshold for non maximum suppression is set to 50 gray levels, and the number of consecutive pixels that need to be detected is set to 9. The number of FPs removed and the associated time are used as the comparison indices. Fig 11 shows the results.

The number of FPs of the 3D image of the kettle found by each algorithm in 3.2 seconds is shown in Fig 11(a). The FPs found by Moravec algorithm, Fast algorithm, Harris algorithm, and the proposed algorithm are 40, 51, 74, and 120 in 3.2 s, respectively. In addition, Fig 11(b) shows the response time of each algorithm to detect 50 FPs of the 3D image of the vase. It can be concluded that the corresponding times of Moravec algorithm, Fast algorithm, Harris algorithm and research algorithm are 1.43s, 2.67s, 1.86s and 0.43s, respectively. Thus, the feature extraction algorithm used for the research has a certain degree of superiority over other algorithms in terms of response time and detection accuracy.

#### 3.1.3 Performance evaluation of curve reconstruction algorithms.

**Fig 12:**
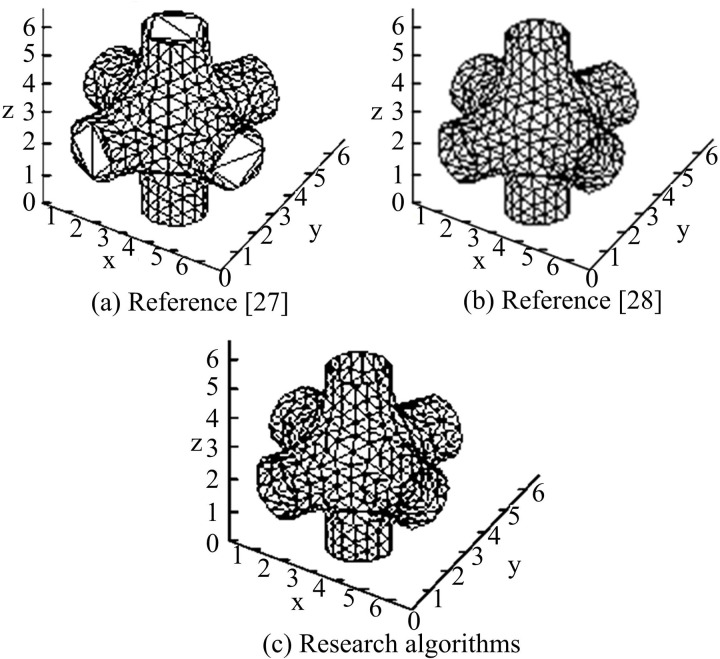
Simulation test results of three curve reconstruction algorithms.

The experimental environment of the test is the same as in Section I. The hybrid tree curve reconstruction algorithm based on literature [27], the 3D curve reconstruction algorithm based on DL from literature [28] and the research algorithm are chosen to test the 3D curve reconstruction model of a mechanical part, and the results are shown in Fig 12.

The three curve reconstruction techniques’ simulation results for the 3D curve reconstruction of mechanical parts are displayed in Fig 12. It is clear that even while the hybrid tree-based curve reconstruction algorithm is easy to use and straightforward, the generated curve frequently falls short of the requirements for a high-quality curve. Although the 3D curve reconstruction algorithm based on DL can generate more complete curves, it involves more parameters, which leads to a high time complexity, limiting its feasibility and efficiency in practical applications. The curve reconstruction algorithm adopted in the study performs Delaunay triangulation on the Bezier curve, which can obtain a more reasonable and accurate curve topology. This can obtain high quality curve reconstruction results and avoid the problem of excessive complexity of the algorithms. The time complexity of the three algorithms for curve reconstruction is quantified to test the time spent by each algorithm for 3DM curve reconstruction of this mechanical part. Table 2 displays the specific outcomes.

**Table 2:** Runtime comparison of three curve reconstruction algorithms.

/		Types of algorithms		
		Literature [27]	Literature [28]	Research algorithms
Comparison of operational efficiency (s)	Triangulation	0.561	0.522	0.327
	Connection time	0.068	0.053	0.032
	Intersection factor time	0.008	0.004	0.003
	Extraction time	0.237	0.279	0.162
	Total time	0.874	0.855	0.524

In Table 2, the total time used by the literature [27] algorithm, the literature [28] algorithm, and the algorithm used in the study to perform 3DM curve reconstruction of mechanical parts is 0.874s, 0.855s, and 0.524s, respectively. This suggests that the study’s reconstruction algorithm has a low time complexity.

### 3.2 3DM interaction design model modeling effect evaluation

**Fig 13:**
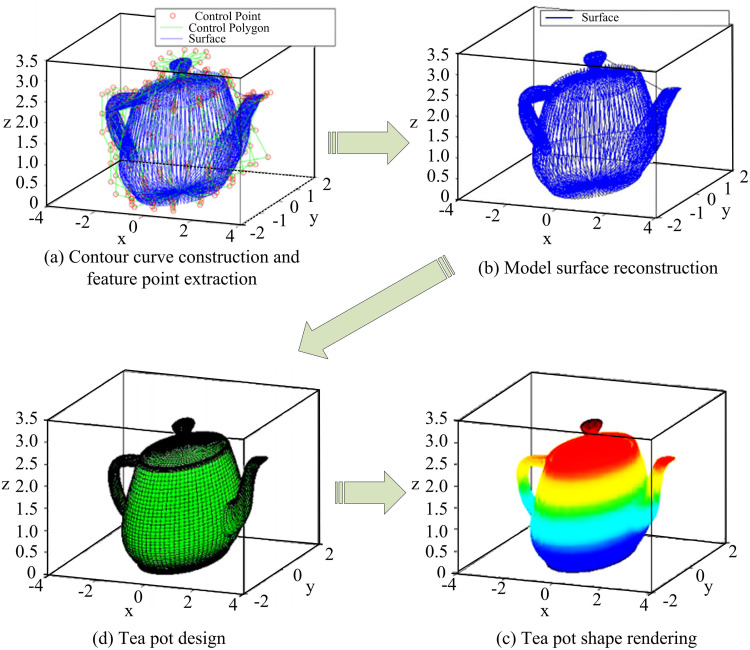
Modeling effect of 3DM interaction design models.

An image of a teapot provided by a customer is used as an experimental object to evaluate the modeling effect of the 3D-MID model constructed in the study, and the results are shown in Fig 13.

**Fig 14:**
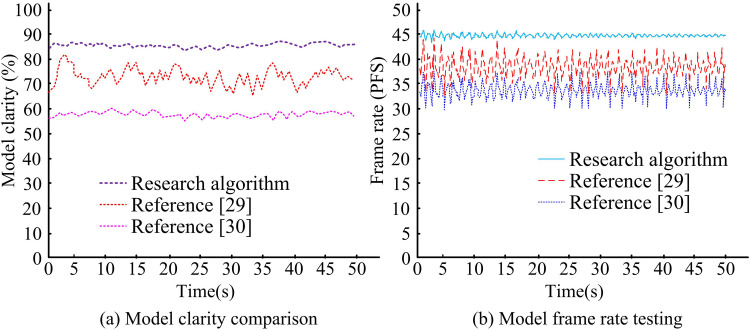
Model sharpness and frame rate test results.

As shown in Fig 14, after the image of the teapot is output, the model extracts the FPs of the teapot and then utilizes spline interpolation to clearly construct the outline of the teapot. Then the shape of the teapot can be adjusted according to the demand, and the teapot can be smoothed and rendered after satisfaction. The study constructed a 3D-MID model to demonstrate its superiority over previous 3DM models. To this end, the study constructed a teapot model using the 3DM models from literature [29] and [30] as a comparison. The model clarity and frame rate test results are displayed in Fig 14.

**Fig 15:**
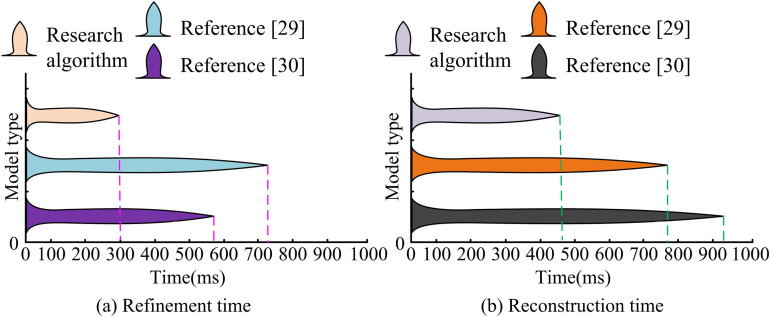
Model refinement time and refactoring time test results.

Fig 14(a) shows the results of the clarity comparison between the three models in the process of constructing the teapot, and the clarity of the model constructed by the study is more stable with time, which is maintained at about 86%. On the other hand, the model constructed by literature [30] is only about 58%, although the clarity is also more stable. The clarity of the model constructed by literature [29] fluctuates more, around 65%-82%. Fig 14(b) displays the results of the comparison of the frame rate changes during the construction of the teapot by the three models. The frame rate of the model constructed by the study has been more stable, around 45 PFS, while the frame rate of the model constructed by literature [29] and literature [30] varies more and has a lower frame rate. It indicates that the 3DM constructed model constructed by the study has stronger performance in terms of stability and clarity. Fig 15 displays the results of a retest of the refinement and reconstruction times for the three approaches used to develop the teapot model.

From Fig 15(a), the refinement time for designing the teapot model in literature [29], literature [30] and the research method is 720ms, 583ms and 300ms, respectively. From Fig 15(b), the reconstruction time for designing the teapot model in literature [29], literature [30] and the research method is 779ms, 918ms and 472ms, respectively. It can be concluded that the research method used the time is the least and is retained at the millisecond level, so the 3D-MID model constructed by the study can fully satisfy the needs of common users and takes less time overall.

## 4. Discussion

3D-MID model building combining Bezier curves and HCDA is an innovative attempt, which is superior in many aspects by accurately identifying the key FPs of an image and using Bezier curves to smoothly depict the contours of 3DM [31,32]. The AMF fusion 3 times discrete wavelet transform denoising algorithm used in the study can effectively denoise the image. The PSNR value of the trucks and APCs image denoising was 43.18. Compared with the other four algorithms under the same conditions, it had good denoising effect. Moreover, it completed the denoising of the modeling image and increase the clarity of the image. At the same time, the CSS technique used in the study to improve the Harris feature detection algorithm can be fast and effective in the extraction of image features. In the experiment, the feature extraction algorithm detected the number of kettle 3D image FPs of 120 in 3.2s, and detected 50 vase 3D image FPs in only 0.43s. In addition, the total curve reconstruction time of a mechanical part model by the Bezier curve fusion Delaunay triangulation curve reconstruction algorithm adopted in the study was only 0.524s. While the hybrid tree curve reconstruction algorithm based on hybrid tree curve reconstruction algorithm and the 3D curve reconstruction algorithm based on DL required 0.874s and 0.855s, respectively. It indicated that the research adopts the reconstruction method could complete the reconstruction task more efficiently and meet the modeling requirements. The 3DM design required high definition of the model image, high frame rate when the platform was running, and the model production time should be as small as possible in order to meet the building requirements of the 3DM platform [33,34]. While the study adopted the building method, the clarity was stable at about 86% throughout the design process, the frame rate was stable at about 45PFS, and the refinement time and reconstruction time took 300ms and 472ms, respectively. It could quickly and accurately realize the 3D design of the target image.

## 5. Conclusion

3D-MID is important in 3D model construction, therefore, this research built a 3D-MID model. The study denoised the incoming and outgoing target images, and then performed image FP detection and obtains the FPs. Based on the obtained FPs, the contour curve was drawn, and then the curve of the image was reconstructed and adjusted and rendered, and finally the 3DMI of the target is output. The research model exhibited high accuracy and stability relative to other model-building methods. Traditional methods often rely on complex mathematical models and high-precision sensor data, whereas the HCDA utilized in the research could accurately identify the FPs of the image, providing an accurate basis for the subsequent 3DM. When combined with Bezier curves, these FPs could be smoothly connected to generate natural and smooth 3DM contours. Secondly, the research model was more efficient than other model-building methods. This was because the model was constructed by identifying key FPs before curve fitting, which could significantly reduce the amount of computation and improve the design efficiency compared to methods that directly perform 3D reconstruction at the pixel level. Finally, the research model was highly interactive, as it allowed the designer to make modifications and adjustments based on the identified FPs. This high degree of interactivity made the design process more flexible and better able to match the designer’s intent and the customer’s needs. The 3D-MID model has shown great potential for application in real-world scenarios, especially in 3DM fields that require high precision, efficiency and interactivity, such as product design, virtual reality, medical image reconstruction, and so on. It can provide more natural and smooth 3D model contours while reducing modeling complexity, speeding up the design process, meeting the diverse needs of designers and customers, and providing important impetus for technological innovation and industrial upgrading in related fields. However, the smoothness of the model’s contour curves could be improved. Subsequent research will address this aspect.

## Supporting information

S1 Data Minimal data set definition. (DOCX)
