# Supplementary material for: Interactive design modeling of 3D styling combining Bezier curve and Harris corner point detection algorithm
Source: PLoS One. 2025 Apr 16;20(4):e0319323. doi: 10.1371/journal.pone.0319323 (PMC12002535; doi:10.1371/journal.pone.0319323)
Supplement: S1 Data — (DOCX) [file pone.0319323.s001.docx]

Minimal Data Set Definition

Fig. 2 Schematic diagram of spline curve adjustment

**The data in Figure 2**

| Parameter | Coordinate | | | | | | | | | |
| --- | --- | --- | --- | --- | --- | --- | --- | --- | --- | --- |
| Parameter 1 | (-4,1) | (-3,0.5) | (-2,1.0) | (-1,3) | (0,2.1) | (1,0.2) | (2,0) | (3,1.1) | (4,2.0) | (5,0) |
| Parameter 2 | (-4,1) | (-3,-1.1) | (-2,2.5) | (-1,3) | (0,1.0) | (1,0.5) | (2,-1.2) | (3,2.8) | (4,2.0) | (5,1.0) |
| Parameter 3 | (-4,1) | (-3,-0.9) | (-2,1.4) | (-1,3) | (0,1.9) | (1,0.2) | (2,0.1) | (3,1.1) | (4,2.0) | (5,0) |
| Parameter 4 | (-4,1) | (-3,-0.5) | (-2,1.5) | (-1,3) | (0,1.9) | (1,0.3) | (2,0.1) | (3,1.7) | (4,2.0) | (5,0) |
| Parameter 5 | (-4,1) | (-3,-0.5) | (-2,1.1) | (-1,3) | (0,2.1) | (1,0) | (2,0.1) | (3,1.0) | (4,2.0) | (5,0) |

Fig. 8 PSNR values for different denoising algorithms

**The data in Figure 8**

| Index | Method 1 | Method 1 | Method 1 | Method 1 | Research algorithms |
| --- | --- | --- | --- | --- | --- |
| PSNR value | 35.12 | 28.02 | 37.68 | 38.28 | 43.18 |

Figure 9 Visual fidelity and noise residual detection results of different denoising algorithms

**The data in Figure 9**

| Index | Method 1 | Method 1 | Method 1 | Method 1 | Research algorithms |
| --- | --- | --- | --- | --- | --- |
| Visual fidelity | 61% | 72% | 64% | 66% | 93% |
| Noise residual | 15% | 18% | 10% | 15% | 3% |

Fig. 11 Comparison of different feature extraction algorithms

**The data in Figure 11**

| Time(s) | Number of feature points | | | |
| --- | --- | --- | --- | --- |
|  | Research algorithm | Moravec | Fast | Harris |
| 0.4 | 40 | 30 | 5 | 18 |
| 0.8 | 43 | 20 | 19 | 20 |
| 1.2 | 46 | 20 | 17 | 20 |
| 1.6 | 61 | 31 | 20 | 32 |
| 2.0 | 65 | 31 | 21 | 65 |
| 2.4 | 71 | 40 | 39 | 61 |
| 2.8 | 83 | 40 | 52 | 71 |
| 3.2 | 120 | 40 | 51 | 74 |
| \ | Research algorithm | Moravec | Fast | Harris |
| Time(s) | 0.43s | 1.43s | 2.67s | 1.86s |

Fig. 14 Model sharpness and frame rate test results

**The data in Figure 14**

| Model clarity (%) | Model | 0 | 5 | 10 | 15 | 20 | 25 | 30 | 35 | 40 | 45 | 50 |
| --- | --- | --- | --- | --- | --- | --- | --- | --- | --- | --- | --- | --- |
|  | Research algorithm | 86.2 | 86.1 | 86.0 | 86.3 | 86.1 | 86.2 | 86.0 | 86.3 | 86.4 | 86.1 | 86.3 |
|  | Reference [29] | 68.3 | 83.1 | 69.2 | 74.2 | 76.4 | 73.5 | 76.1 | 72.3 | 74.2 | 75.4 | 72.2 |
|  | Reference [30] | 58.3 | 59.3 | 58.4 | 59.4 | 58.6 | 57.9 | 58.6 | 59.2 | 57.6 | 58.3 | 59.4 |
| Frame rate (PFS) | Model | 0 | 5 | 10 | 15 | 20 | 25 | 30 | 35 | 40 | 45 | 50 |
|  | Research algorithm | 45.3 | 44.9 | 45.2 | 44.8 | 45.2 | 44.7 | 45.1 | 44.9 | 45.2 | 45.3 | 44.9 |
|  | Reference [29] | 35.6 | 44.5 | 37.2 | 43.5 | 36.2 | 44.5 | 35.1 | 43.4 | 36.2 | 44.2 | 35.2 |
|  | Reference [30] | 36.1 | 30.2 | 37.3 | 31.2 | 35.2 | 30.8 | 36.2 | 31.2 | 35.2 | 30.5 | 34.2 |

Fig. 15 Model refinement time and refactoring time test results

**The data in Figure 15**

| Index | Research algorithm | Reference [29] | Reference [30] |
| --- | --- | --- | --- |
| Refinement time | 300ms | 720ms | 583ms |
| Reconstruction time | 472ms | 779ms | 918ms |
